# Changes in the Stress Response and Fitness of Hybrids Between Transgenic Soybean and Wild-Type Plants Under Heat Stress

**DOI:** 10.3390/plants14040622

**Published:** 2025-02-19

**Authors:** Li Zhang, Qi Yu, Xin Yin, Laipan Liu, Zhentao Ren, Zhixiang Fang, Wenjing Shen, Shengnan Liu, Biao Liu

**Affiliations:** 1 Key Laboratory on Biodiversity and Biosafety, Nanjing Institute of Environmental Sciences, Ministry of Ecology and Environment, Nanjing 210042, China; feiniao8897@126.com (L.Z.); 15968821722@163.com (Q.Y.); njfu_shin@163.com (X.Y.); liulaipan@163.com (L.L.); rztkkk@163.com (Z.R.); zxfang23@126.com (Z.F.); swj@nies.org (W.S.); 2 Institute of Plant Protection, Sichuan Academy of Agricultural Science, Chengdu 610066, China

**Keywords:** genetically modified soybean, transgene escape, F_2_ hybrid, high temperature, competition

## Abstract

Understanding the ability of hybrids of genetically modified (GM) soybean and wild soybean to survive and reproduce under unfavorable conditions is critical for answering questions regarding risk assessment and the existence of transgenes in the environment. To investigate the effects of high-temperature stress on soybean growth and competitive ability, the GM soybean DBN8002, which expresses the VIP3Aa and PAT proteins, and F_2_ generations derived from a cross between GM soybean and NJW (wild soybean) were placed in a greenhouse with an elevated temperature (38/32 °C) for 14 days, and the plant agronomic performance and foreign protein levels of hybrid soybean were evaluated to observe their responses to high temperature. The results revealed that the VIP3Aa and PAT protein levels in F_2_ and GM were not influenced by high-temperature stress. In contrast, the pollen germination, pod number, hundred-seed weight, and seed vigor of the F_2_ hybrid and parent soybean plants decreased after high-temperature stress. However, except for the number of fully filled seeds per plant, the above parameters of the F_2_ hybrid were similar to or slightly lower than those of wild soybean, and no significant difference in fitness was observed between the F_2_ hybrid and wild soybean, indicating that the growth and competitive ability of the hybrid were similar to those of its female parent under heat stress conditions, resulting in the transgenes persisting and spreading within agricultural ecosystems. Our results enhance the understanding of the GM soybean plant’s response to heat stress, lay the foundation for breeding heat-resistant soybean varieties, and provide new insights and advanced information on the ecological risks arising from the escape of transgenes.

## 1. Introduction

Genetically modified (GM) soybeans were one of the earliest introduced GM crops for commercial cultivation and rank first in terms of planting area worldwide [1]. By 2019, GM soybean plants with beneficial traits, such as insect resistance, herbicide tolerance, and salt tolerance, were extensively planted in 29 countries, resulting in important economic and social benefits [1,2]. However, GM soybean plants have not been officially commercialized to date in East Asian countries such as China, Japan, and Korea. There are two limiting factors for the application and commercialization of GM soybean cultivation in these countries. The first is poor public acceptance of transgenic technology, mainly because of concerns about the potential adverse effects of GM soybeans on human and animal health [3,4,5,6]. Another significant problem is gene flow via seeds during transportation or the pollen-mediated gene transfer of transgenic soybean plants to their wild relatives, which could result in resistant weeds and a series of environmental issues [7,8,9].

The wild resource of cultivated soybeans, *Glycine soja*, is naturally distributed in East Asia, and China is a center for wild soybean cultivation [10,11,12]. Wild soybeans are widely distributed in 31 provinces and the autonomous regions of China [13]. Since cultivated soybeans and wild soybeans both belong to the genus *Soja*, they can cross freely and produce fertile hybrid offspring [14]; therefore, wild soybean is commonly used as an important genetic resource for soybean breeding. After GM soybean application, GM soybean and its transgenes can naturally spread via seeds during transportation and through pollen-mediated gene transfer to wild soybean plants [15,16]; if this occurs, the genetic introgression of foreign genes could contribute to the loss of genetic integrity in wild soybean. However, hybrids often show higher growth rates and greater fecundity than the parental lines due to heterosis [17,18], and they can rapidly accumulate and disseminate in weedy and wild populations, which may lead to a decline in or even the extinction of wild soybean populations [19,20].

Recently, some researchers have focused on the ecological impact of gene flow on wild soybean populations. Yook et al. [21] reported that hybrids between glufosinate-resistant (GR) soybean and wild soybean (IT182932) had similar numbers of pods and seeds to wild soybean, indicating that transgenes of the GR soybean might disperse into wild populations and persist in the agroecosystem due to the relatively high fitness of the hybrid progeny. Pot-based experimental studies conducted by Zhang et al. [8] revealed that F_2_ and F_3_ hybrids between the Roundup Ready soybean and wild soybean exhibited lower seed germination rates and higher seed productivity than the GM soybean, indicating that the adaptability of hybrids may increase the possibility of dispersal of transgenes in wild soybean relatives. Guan et al. [22] and Liu et al. [23] reported no obvious decline in the fitness of hybrids and wild-type soybeans. The above studies were conducted mainly under suitable growing conditions and well-resourced cultivation practices. However, soybean plants are usually challenged by various abiotic stressors during their growth cycles, such as cold, drought, and salt stress, which can directly or indirectly influence plant fitness via their effects on survival, growth, and reproduction. Nevertheless, few studies have been conducted to evaluate the competitive ability of hybrids under stressful environmental conditions.

In recent years, extreme high-temperature events have occurred increasingly frequently worldwide [24,25,26], severely affecting normal crop growth and production. Many studies have shown that the high-temperature stress caused by global warming has led to pollen abortion, kernel shrinkage, and reduced pod setting and seed weight [27,28,29], which has become an increasingly serious problem and a limiting factor in soybean production [30,31,32,33]. Compared with cultivated soybean plants, wild soybean plants are generally more resistant to abiotic stressors such as extreme temperatures, drought, and salt stress [34,35,36]. For example, Veremeichik et al. [37] reported that 35 days of cold (16/12 °C) and heat (36/34 °C) treatments did not affect the growth and biomass accumulation of wild soybean compared with cultivated soybean. Thus, the vegetative and reproductive growth abilities of crop-wild hybrid soybean plants under relatively high temperatures cannot be effectively predicted. If hybrids have low tolerance to high-temperature stress, their growth and reproductive abilities will be adversely affected by relatively high temperatures. In contrast, if the tolerance of the hybrid to high temperatures is similar to that of the female parent or if the hybrid is even more resistant to high temperatures (i.e., hybrid vigor), this would indicate that these heat-resistant genes in wild soybean plants are effectively utilized to enhance resistance through introgression breeding, which may considerably increase the competitive ability of the hybrid in the agroecosystem.

GM soybeans have consistently been the focus of research and development related to GM technology in China [38]. Since 2008, the government has implemented and successfully conducted the GMO Special Project [39,40], and independent innovation capacity in this field has significantly increased in recent years. By 2024, numerous GM soybean varieties were developed and showed substantial application prospects in agriculture, and three GM soybean varieties were issued with biosafety certificates by the Chinese government. One of these certificates was issued for DBN8002 soybean, which is highly resistant to lepidopteran pests and tolerant to glyphosate [41], demonstrating strong potential for commercialization. In 2023, DBN8002 was approved for future application in the Huang-Huai-Hai region of China. The region has a vast land area and abundant resources, with rain and high temperatures in summer. In recent years, extremely high temperatures have occurred frequently in the region during summer (July–August) [42,43], which severely affects the yield and quality of soybean [30,31,32]. However, little is known about the competitive ability of GM soybean and hybrid offspring under heat stress. In this study, we analyzed the seed vigor, foreign gene expression, and plant growth performance of GM, wild, and hybrid populations under heat stress conditions, to evaluate how different soybean accessions respond to heat stress, provide a theoretical basis for the breeding of heat-resistant GM soybean crop varieties in the future, and offer further insight to understand the risk of transgene escape from GM soybean to its wild relative.

## 2. Results

### 2.1. In Vitro Pollen Germination Analysis

Compared with the control, high temperatures during the flowering and pod formation periods significantly decreased the pollen germination rates of GM soybean, F_2_(+), and F_2_(−) plants by 35.57%, 35.45%, and 31.03%, respectively (Table 1). In addition, the pollen germination rates were reduced in the wild soybean groups, but the germination rate did not significantly differ from that of the control pollen.

Additionally, under the same conditions, no significant difference in pollen viability was detected among the different soybean materials.

### 2.2. Aboveground Biomass

**Figure 1:**
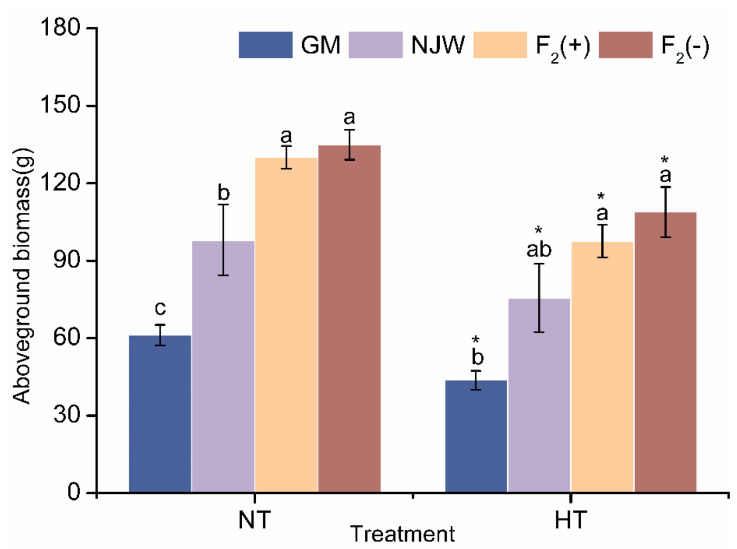
Mean biomasses of GM, wild, and hybrid soybean plants under normal and temperature-stress conditions. The values are presented as the means ± standard errors of the means (SEs) (*n* = 10 in each group). The same letter indicates a nonsignificant difference and different letters indicate a significant difference (*p* < 0.05). Asterisks indicate significant differences between control and high-temperature treatment specimens (* for *p* < 0.05). NT, normal temperature treatment; HT, high-temperature treatment; GM, genetically modified; NJW, Nanjing wild soybean; F_2_(+), F_2_ plants with foreign genes; F_2_(−), F_2_ plants negative for foreign genes.

Under control conditions, the F_2_ hybrid plants had the greatest biomass, at 134.87 and 130.02 g for F_2_(−) and F_2_(+), respectively, followed by the wild-type soybeans, and the genetically modified soybeans had the lowest biomass. There was a significant difference among the three groups (Figure 1).

After heat stress treatment, the aboveground biomass of the F_2_ generation (108.81 g and 97.62 g) was slightly greater than that of the wild-type soybeans (75.55 g) and significantly greater than that of the GM-type soybeans (43.71 g), and there were no significant differences in aboveground biomass between the F_2_ population and the wild-type soybeans. In addition, there were varying degrees of aboveground biomass decrease in all four soybean varieties after heat stress. Among them, significant reductions in biomass were found for GM and F_2_(+), with decreases of 28.54% (from 61.17 to 43.71 g) and 24.92% (from 130.02 to 97.62 g), respectively. Compared with that of the control, the biomass of NJW and F_2_(−) decreased by 18.78% and 19.32%, respectively, with no significant differences.

### 2.3. Fecundity

#### 2.3.1. Number of Pods per Plant

Compared with the control treatment, heat stress significantly reduced the number of pods per plant. The number of pods of F_2_(+) and F_2_(−) decreased from 645.60 to 380.90 and from 619.90 to 401.40, significantly decreasing by 41% and 35.25%, respectively (Table 2). Similarly, the number of pods on NJW and GM soybean plants decreased after high-temperature treatment, with decreases of 22.30% and 21.94%, respectively, this being significantly different from those on the control.

Analysis of variance revealed that there was no significant difference in the number of pods per plant between the hybrid F_2_ plants and the wild soybean parent under control conditions or heat-stress conditions.

#### 2.3.2. Seed Number and Full Seed Number per Plant

Table 3 shows that the seed number and the full seed number per plant decreased significantly in GM, F_2_(+), and F_2_(−) hybrid soybean plants after 14 days of heat stress. The seed numbers decreased from 81.30, 944.3, and 919.50 to 46.40, 520.20, and 447.50, respectively. In particular, F_2_ (+) showed the greatest decrease (52.56%). Moreover, the seed number and full seed number in wild soybean plants also decreased but not significantly.

Under both control and heat-stress conditions, both the seed number and full seed number were highest in the wild soybean seeds and lowest in the GM soybean seeds. The seed number and full seed number of the F_2_ hybrid seeds were similar to those of the wild-type soybean plants in the control group; however, significant differences were observed in the seed number and full seed number between the F_2_(+) and wild-type soybean plants under heat stress.

#### 2.3.3. One-Hundred-Seed Weight and Seed Weight per Plant

In both the control and heat-stress treatments, the 100-seed weights were highest in the GM soybean seeds and lowest in the wild-type soybean seeds, and the F_2_(+) and F_2_(−) hybrids presented higher 100-seed weights than their wild-type soybean counterparts did; no parameters significantly differed between the F_2_(+) and F_2_(−) populations (Table 4). After 14 days of heat-stress treatment, the 100-seed weight per plant decreased significantly in GM and F_2_(+) and F_2_(−) hybrid soybean plants but did not significantly decrease in wild soybean plants.

The F_2_ hybrids presented greater seed weights than the wild-type soybean plants under both normal and high-temperature conditions, and the difference was significant (*p* < 0.05) under normal conditions. After high-temperature treatment, seed weight also significantly decreased in all the soybean varieties, with significant decreases of 52.88%, 50.32%, 48.33%, and 44.13% for F_2_(−), F_2_(+), GM, and wild soybean, respectively, as shown in Table 4.

### 2.4. Vip3Aa and PAT Protein Contents in Soybean Leaves

An ELISA was performed to quantify the levels of PAT and Vip3Aa proteins in different soybean leaf samples. The results revealed that the wild soybean, F_2_(−), and control samples were negative for PAT and Vip3Aa expression, whereas foreign proteins were detected in all the GM and F_2_(+) leaf samples.

**Figure 2:**
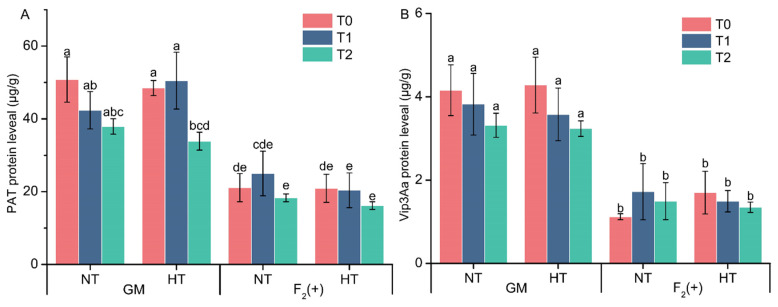
Changes in the expression levels of PAT (**A**) and Vip3Aa (**B**) in the leaves of two soybean varieties before treatment (T_0_), after treatment (T_1_), and 7 days after treatment (T_2_) under normal conditions and high-temperature conditions. The values are presented as the mean ± standard errors of the means (SEs). The same letter indicates a nonsignificant difference and different letters indicate a significant difference (*p* < 0.05). NT, normal temperature treatment; HT, high-temperature treatment; GM, genetically modified; NJW, Nanjing wild soybean; F_2_(+), F_2_ plants with foreign genes; F_2_(−), F_2_ plants negative for foreign genes.

In both the control and high-temperature treatment samples, the PAT and Vip3Aa contents in the leaves of the GM soybean plants were, on average, greater than those in the leaves of the F_2_(+) soybean plants (Figure 2). There were no significant differences in the foreign protein contents between the treatments, and both the PAT and Vip3Aa contents were similar in the plants grown under control and high-temperature conditions.

### 2.5. Seed Vigor

In both the control and high-temperature treatments, the total germination rate of the wild-type soybean plants was the highest, followed by that of the F_2_ hybrids and, finally, the GM soybean plants (Table 5). The seed germination rates of the NJW and F_2_(+) hybrid seeds were slightly higher than those of the GM and F_2_(−) hybrid seeds in the control samples, and the seed germination rates of the NJW and F_2_(−) hybrid seeds were significantly higher than those of the GM and F_2_(+) hybrid seeds under high-temperature treatment. In addition, no significant differences were detected between F_2_(+) and F_2_(−) hybrid seeds.

After 14 days of heat treatment, the seed germination rates greatly varied among the soybean materials. The germination rates of the NJW and F_2_(−) seeds were 84.50% and 72.50%, respectively, which were greater than those of the GM and F_2_(+) seeds (46.50% and 58.00%, respectively). Moreover, a significant decrease in seed germination was recorded in the NJW, F_2_(−), F_2_(+), and GM soybean seeds after high-temperature treatment compared with the control seeds; however, the decrease was not large in the NJW and F_2_(−) samples, at 10.58% and 16.18%, but was greater for the GM and F_2_(+) samples at 46.24% and 34.83%, respectively. Additionally, the percentage of dead seeds increased significantly in all soybean materials after heat-temperature treatment, especially in the GM and F_2_(+) hybrid seeds; approximately 53.5% and 42% of the GM soybeans and F_2_(+) hybrid seeds failed to germinate (they had generally rotted or mildewed), respectively, during the test.

### 2.6. Relative Composite Fitness

**Figure 3:**
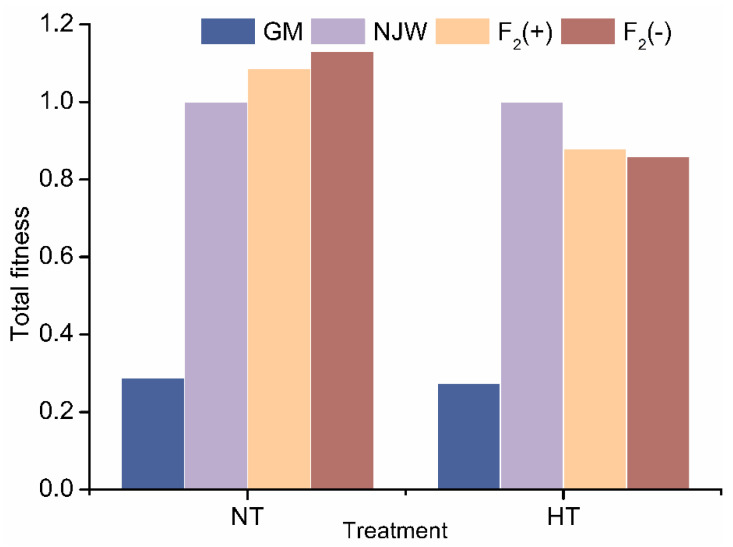
Composite fitness of F_2_ hybrids and wild-type soybeans under normal and high-temperature conditions. NT, normal temperature treatment; HT, high-temperature treatment; GM, genetically modified; NJW, Nanjing wild soybean; F_2_(+), F_2_ plants with foreign genes; F_2_(−), F_2_ plants that were negative for foreign genes.

Under normal growth conditions, the fitness values of the F_2_(+) and F_2_(−) hybrids were 1.07 and 1.15, respectively, both of which values were greater than those of the wild-type soybean parent (Figure 3). In contrast, the fitness of F_2_(+) and F_2_(−) hybrids was lower than that of their wild soybean counterparts. However, no differences in relative composite fitness were detected between the hybrid and the wild soybean, either in the control samples or under high-temperature conditions.

## 3. Discussion

Whether the escape of foreign genes from GM soybeans poses potential ecological risks largely depends on the fitness of the hybrid offspring [44,45,46]. Generally, the higher the level of hybrid fitness is, the greater the risk of transgene escape and transmission [47,48]. In addition to the parental genome, environmental stress related to soil conditions, insect pressure, and climatic conditions can affect plant fitness [49,50,51]. Thus, we evaluated the ecological risk of GM and its hybrid soybean under heat-stress conditions to gain a comprehensive understanding of the ecological risk of planting GM soybeans, supplementing previous research in this field of study.

### 3.1. Effects of High-Temperature Stress on the Aboveground Biomass of Soybean

Aboveground biomass is not only an important predictor of crop growth status and yield but is also an indicator of plant competitive ability and biotic resistance [52,53,54,55]. In the present study, biomass decreased in all three soybean varieties; however, although there was a significant decrease in biomass in the F_2_ soybean, its biomass was still greater than that of wild soybean after high-temperature stress, suggesting that the F_2_ hybrid still had high vegetative growth at high temperatures. In a two-year agricultural field study, Yook et al. [21] found that hybrids showed similar characteristics to wild soybeans in terms of above-ground biomass. Zhang et al. [8] and Liu et al. [23] also obtained similar results under controlled conditions. The increase in F_2_ soybean biomass may be beneficial for individual competitiveness and organizational performance. Notably, Veremeichik et al. [37] reported that long-term (35 days) heat (36/34 °C) treatments did not affect growth and biomass accumulation in wild soybeans, whereas a significant decrease in biomass was observed in two cultivated soybeans. These findings are consistent with the results of this study, indicating that wild soybeans may be more tolerant to heat stress conditions than GM cultivated soybeans.

### 3.2. Effects of High-Temperature Stress on Pollen Viability and Soybean Fecundity

During the period of anthesis, heat stress causes pollen deformity and anther indehiscence, inhibits pollen-tube growth, decreases pollen viability, and ultimately leads to a reduction in soybean yield [56,57]. Similar phenomena were observed in this study; a decrease in pollen viability was observed in all soybean materials after heat stress, with the greatest decrease occurring in the F_2_(−) plants, which was close to 35.5%, whereas the F_2_(+) plants presented the lowest pollen viability. Nevertheless, no significant differences in pollen germination were detected among the four plant materials, suggesting that the influences of high-temperature stress on the pollen from different soybean materials were roughly the same. To date, only a few researchers have evaluated changes in the viability of GM soybean or hybrid pollen under stress conditions. Liang et al. [58] reported that weed competition or barren soil can reduce the pollen germination rate of wild soybeans and the two hybrid offspring, but no difference was found in the pollen germination rate between hybrid and wild parents under the same planting conditions. In the present study, similar conclusions were reached.

After heat stress, both parent plants and hybrid plants presented significant decreases in the numbers of pods and seeds, but these two parameters did not differ between the F_2_ hybrid and the wild-type parent. Like in our study, in a two-year pot-based experiment, Liang et al. [58] reported that F_2_ hybrids between herbicide-tolerant transgenic soybeans (T14R1251-70) and wild soybeans produced more pods and full seeds than did wild soybeans under weed-stress conditions. Our findings and those of previous studies suggest that hybrids possess greater reproductive ability, even under unfavorable conditions. In addition, previous results have shown that F_2_ and F_3_ hybrids between GM and wild soybeans have larger and heavier seeds [8,21,46]. In the present study, the F_2_ hybrid plants presented larger seeds and greater 100-seed weights than did the wild-type plants, which may also explain why the seed weight was greater in the F_2_ hybrid soybean than in the wild-type soybean plants in this study. To date, several studies have been conducted to assess the effects of heat stress on soybean field performance, with a particular focus on cultivated soybeans. These studies showed that a few days of plant exposure to high temperatures (35 °C or above) during the growth period can cause large yield losses through delays in flowering, flower and pod abortion, and a reduction in the 100-seed weight [29,41,59,60,61]; their findings are similar to the conclusions of this study.

### 3.3. Effects of High-Temperature Stress on Vip3Aa and PAT Protein Contents in Soybean Leaves

After two weeks of heat stress, the foreign genes were expressed normally in the GM- and F_2_-positive soybean plants and their expression levels differed little from those in the control plants, suggesting that high temperatures at the R3 stage had little or no effect on foreign gene expression levels. To date, few studies have investigated changes in foreign proteins in GM crops and hybrid plants under heat stress. Trtikova et al. [62] investigated the expression levels of the Bt protein in the leaves of two transgenic maize varieties, namely, *Bt* PAN 6Q-321B (white Bt maize) and *Bt* PAN 6Q-308B (yellow Bt maize), under high-temperature stress (21–30 °C, with a maximum of 45 °C at noon), and their results revealed that high temperature had little effect on the Bt protein level. A similar result was reported by Chen et al. [63], who reported that the Cry1Ac contents in the leaves of Sikang1 and Sikang3 cotton plants under short-term high-temperature stress (37 °C for 24 h) were not significantly different from those under optimal conditions. However, Zhang et al. [64] reached the opposite conclusion; they reported a significant decrease in the Cry1Ac protein content after *Bt* cotton was exposed to 38 °C continuously for 24 h. Our findings and the abovementioned results suggest that foreign gene content is not only influenced by the genetic background of a given plant variety but also by stress-inducing temperatures and exposure time. Under stressful conditions, the concentration of foreign protein is highly variable and is, therefore, difficult to predict [62].

### 3.4. Effects of High-Temperature Stress on Seed Vigor

Seed vigor is an important indicator of seed germination, seedling growth potential, plant stress resistance, and production potential [65]. The results of the seed germination tests demonstrated that both the GM, wild, and F_2_ hybrid seed germination rates significantly decreased after high-temperature treatment. In the present research, visual evidence of seed damage, such as incomplete filling, wrinkling, cracking, and sunken lesions was more common in the high-temperature treatment group than in the control group (Figure S2). Shu et al. [66] and Liu et al. [67] reported that high temperatures result in damage to seeds, including stress cracks, shrinkage, and decreased seed germination [68,69], which is consistent with our observations. Such damage caused by heat stress may increase the risk of seed mold infection, leading to a decrease in seed germination and emergence rates.

The germination rates of both parent and hybrid seeds decreased significantly under heat-stress conditions. However, the decreases in the germination rates of the F_2_(+) plants and GM soybean seeds were much greater than those of the wild-type and F_2_(−) plants. A sharp decrease in germination was observed for the F_2_(+) plants and GM soybean seeds, for which the germination rates decreased by 58.0% and 46.5%, respectively, whereas the germination rates of the NJW and F_2_(−) plants were 16.18% and 10.58%, respectively, compared with those of the control plants. In addition, the germination rates of the wild-type soybean and F_2_(−) plants were much greater than those of the GM and F_2_(+) plants. Our results indicate that high temperature may have a greater effect on F_2_(+) and GM soybean seeds than on NJW and F_2_(−) seeds.

In this study, GM soybean plants produced large yellow seeds, whereas F_2_(+) and F_2_(−) had medium-sized seeds that were colored light green and black, respectively (Figure S2). In contrast, wild soybean plants produced small, blackish seeds. Some studies have shown that the seed coat’s color, thickness, and structure both directly and indirectly affect seed germination [70]. Zhang et al. [46] reported that the physical characteristics of the seed coats of F_3_ plants were closely related to seed germination; the darker-colored (brown and black) seeds of the F_3_ hybrid between GM soybean and wild soybean presented a large area of honeycomb-like surface deposits and a thick palisade layer and had a low germination rate and greater overwintering ability than did lighter-colored (yellow-green) F_3_ seeds. Ma et al. [71] and Santos [72] also suggested that soybean seeds with dark seed coats (black and brown) have more agronomic advantages than those with yellow seed coats, such as greater coat thickness, lower permeability, and greater resistance to mechanical damage. In addition, previous studies have demonstrated that dark seed coats have relatively high concentrations of anthocyanins [73], phenolics [74,75], and flavonoids [76,77], and that these substances may increase the capacity of seeds to cope with abiotic stressors [78]. Therefore, the higher germination rates of NJW and F_2_(−) seeds after high-temperature stress compared to those of GM and F_2_(+) soybeans may be related to the coat structure and chemical composition of the seeds, but the specific reasons need to be studied further.

To our knowledge, this is the first report of fitness changes in F_2_ hybrids between genetically modified soybeans and wild soybeans after high-temperature stress. The findings described in this study indicate that high-temperature stress ultimately resulted in reduced fitness in all soybean materials, especially the GM soybean. However, although the average fitness of the hybrids was lower than the fitness of the wild-type relative, no difference was observed in total fitness between the two, indicating that the survival and competitive ability of hybrids are similar to those of their female parents under high-temperature stress conditions. Moreover, it was found that high temperatures did not seem to affect the expression levels of foreign proteins, which could contribute to the ability of hybrid plants to cope with pest stressors. Gompert et al. [79] and Lu et al. [47] suggested that plants with heterosis/hybrid vigor are more able to adapt to more extreme conditions than their parental genotypes, especially in later generations. Thus, continuing to perform studies on fitness changes in later-generation hybrids under stress conditions might contribute to a more comprehensive and systematic understanding of the ecological risks of hybridization.

In addition, in this study, as the hybrids were generated using wild soybean as the pollen recipient, we speculate that the significantly increased fitness of the F_2_ hybrid compared with the GM was mostly due to maternal inheritance. Therefore, searching and mining the related heat tolerance genes from wild soybean plants will be critical to soybean cultivation and food production in the future under global climate warming conditions.

## 4. Materials and Methods

### 4.1. Plant Materials

The GM soybean DBN8002, which is highly toxic to Lepidopteran pests such as *Spodoptera frugiperda* and *Spodoptera exigua* and is tolerant to herbicides [41], can be transformed with the *Vip3Aa* and *PAT* genes [80]. Wild soybean plants were collected from Jiangpu County, Nanjing, Jiangsu Province, China. The seeds of both soybean types were provided by Beijing DaBeiNong Science & Technology Group Co., Ltd. (Beijing, China).

To obtain F_1_ hybrid offspring, the GM soybean DBN8002 was used as a male parent (pollen donor), and wild soybean was used as a female parent (pollen recipient). Artificial pollination was conducted during the flowering season in 2019. In the next year, F_1_ seeds from the cross were collected and selfed to generate the F_2_ generation.

As F_2_ lines are a segregating population, two traits segregate the F_2_ population: the transgenic-positive plants contain foreign genes, whereas the nontransgenic plants do not. To confirm the expression of the foreign gene, the presence or absence of the foreign gene in F_2_ soybean plants was identified using a QuickStix Kit for PAT (EnviroLogix, Portland, ME, USA) and VIP3Aa (EnviroLogix, Portland, ME, USA). Among the 176 plants in the F_2_ population, 42 plants were negative for foreign genes, marked as F_2_(−), whereas 134 plants were positive for foreign genes, marked as F_2_(+). The test results are shown in Supplementary Figure S1.

### 4.2. Plant Growth and Heat-Stress Treatment

In 2022, Nanjing city in China experienced abnormally high temperatures; the average maximum and minimum temperatures in August were 34 °C and 27 °C, respectively, and extremely high temperatures (38 °C or above) lasted for 12 days, with an extremely high temperature of 41 °C. High temperatures in August at the flowering and pod-setting stages (mid-July to mid- to late August) strongly affect the normal growth and development of soybean plants and lead to decreased yield and quality in the field. Thus, to investigate the sensitivity of GM soybean plants, especially hybrid soybean plants, to heat stress, we designed and conducted the current study, and the treatment temperatures were set at 38/32 °C (day/night); 38 °C and 32 °C were the average day and night temperatures during high-temperature weather in August 2022, respectively. In addition, the treatment time and soybean growth period during which these high temperatures occurred were set according to the actual field conditions in Nanjing in the summer of 2022. Because field trials are currently limited by policies in China, the study was conducted under controlled conditions.

Pot-based experiments were conducted at the Nanjing Institute of Environmental Sciences. GM, wild soybean plants, and F_2_ hybrids were given the high-temperature treatment. In 2023, a total of 200 F_2_ seeds, together with 80 seeds each from the parent plants, were sown in seedling trays and incubated in a greenhouse under controlled conditions (25–30 °C, with a day length of 12 h). After 40 days of growth, 50 parent plants and 176 F_2_ seedlings were transplanted into cultivation pots containing soil or nutrient-enriched soil (1:1, *v*:*v*), with 1 seedling per pot. When both flowers and young raised pods (0.5 to 2 cm in length) were present in the soybean plants (the R3 growth stage, according to Fehr et al. [81], 20 plants were randomly selected from each soybean material and moved to a greenhouse for heat stress treatment at 38/32 °C (day/night) for 2 weeks (high-temperature treatment, or HT), while the remaining plants were kept in greenhouse chambers as the controls (normal temperature treatment, or NT). The NT treatment plants were grown in a 16 h light/8 h dark cycle at temperatures of 28 °C during light periods and 18 °C during darkness, with 35–70% humidity. After heat treatment, all the plants were allowed to grow under normal conditions until harvest. In addition, to prevent seed loss due to pod shattering, 1 mm nylon fabric was used to cover each plant when the color of the pods changed from green to brown, and manual weeding and pesticide application were performed as necessary during the experiment.

### 4.3. Effects of High-Temperature Stress on the Growth and Reproduction of Soybean Plants

#### 4.3.1. In Vitro Pollen Germination Rate

Pollen germination was determined using an optimized solid medium, as described previously [23]. At the end of the heat stress treatment, mature pollen from 5 plants was randomly selected and scattered on solid media, and pollen germination was observed under a microscope after 30 min of incubation in a chamber at 28 °C and 100% relative humidity in the dark. For each Petri dish, four replicates were performed in three random visual fields (with approximately 100 pollen grains in each field). Pollen was recorded as having germinated when the pollen tube length exceeded the diameter of the pollen grain [82], and the pollen germination rate was calculated as the percentage of germinated pollen grains among the total number of pollen grains.

#### 4.3.2. Aboveground Biomass and Fecundity

At harvest, 10 plants of each soybean material were randomly chosen, cut at a point 5 cm above the ground, air-dried, and weighed via a weighing balance (PB602-N, Mettler Toledo, Greifensee, Zürich, Switzerland). After the biomass was recorded, fecundity traits such as the number of pods, number of seeds, number of full seeds, 100-seed weight, and seed weight were recorded per plant.

#### 4.3.3. Vip3Aa and PAT Protein Contents in Soybean Leaves

To assess whether heat stress might affect the expression of Vip3Aa and PAT proteins in GM and F_2_ plants, a commercial double-antibody sandwich ELISA kit (enzyme-linked immunosorbent assay) was used to determine the concentration of proteins in soybean leaves. Leaf samples of the same plants from GM, wild soybean, and F_2_ offspring were collected before treatment (T_0_), after treatment (T_1_), and 7 days after treatment (T_2_). The collected leaves were quickly frozen in liquid nitrogen and stored at −20 °C until analysis.

For foreign protein determination, 10~12 mg of leaf tissue was ground in liquid nitrogen, extracted in 1 mL PBS buffer (provided with the kit), and centrifuged for 15 min at 3000× *g*. The supernatant was analyzed for protein content using quantitative ELISA kits (QuantiPlate™ Kit for PAT/bar, Envirologix, Inc., Portland, ME, USA; QuantiPlate™ Kit for Vip_3_A, Youlong Biological Engineering Co., Ltd., Shanghai, China). The subsequent steps were performed according to the instructions provided in the kit manual. The optical density values of the samples were obtained via a microplate reader (EnviroLogix, Portland, ME, USA) at 450 nm, and all the samples were measured in triplicate.

#### 4.3.4. Seed Vigor

To investigate the effects of extremely high temperatures on seed viability, seeds harvested from heat-treated/control GM, wild-type, F_2_(+), and F_2_(−) plants were subjected to germination tests. Prior to the germination test, the seeds of wild and F_2_ soybean plants were carefully scarified by nicking the seed coat with a scalpel to break seed dormancy. For each soybean material, four replicate groups with a total number of 200 seeds (4 × 50 seeds) were prepared. Seeds were placed in Petri dishes containing two layers of moist filter paper and incubated in an incubator (Binder KBF720, Binder Instrument Co., Ltd., Tuttlingen, Baden-Wuerttemberg, Germany) at 25 °C with a 12 h photoperiod and 55% relative humidity for 14 days. The number of germinated seeds was counted daily, and the seeds were subsequently removed from the Petri dishes. Seeds were considered germinated when the radicle length exceeded 2 mm.

### 4.4. Statistical Analysis

SPSS 20.0 statistical software was used for data analysis. The data are expressed as the mean ± standard deviation (x ± SE). A one-way analysis of variance (ANOVA) and Tukey’s multiple comparison test were used for comparisons, and a *t*-test was used to compare the high-temperature treatments with the controls. For all statistical tests, *p* < 0.05 was considered to indicate statistically significant differences.

## Figures and Tables

**Table 1:** Pollen germination rates of GM, wild, and hybrid soybean plants under normal conditions and under high-temperature stress.

Material	Pollen Germination Rate (%)	
	Control	High Temperature
GM	74.5 ± 3.77 a	48.0 ± 4.69 a*
NJW	73.5 ± 3.59 a	59.0 ± 1.73 a*
F_2_(+)	72.5 ± 1.71 a	50.0 ± 3.65 a*
F_2_(−)	77.5 ± 2.63 a	50.0 ± 3.36 a*

Notes: Asterisks indicate significant differences between control and high-temperature treatment materials (* for *p* < 0.05). Different lowercase letters in a column represent statistically significant differences between different materials (*p* < 0.05). GM, genetically modified; NJW, Nanjing wild soybean; F_2_(+), F_2_ plants with foreign genes; F_2_(−), F_2_ plants negative for foreign genes.

**Table 2:** Effects of high temperature on the number of pods per plant of GM, wild, and hybrid soybeans.

Material	Number of Pods per Plant	
	Control	High Temperature
GM	43.30 ± 2.35 b	33.80 ± 2.28 b*
NJW	630.50 ± 33.45 a	489.90 ± 56.07 a*
F_2_(+)	645.60 ± 39.17 a	380.90 ± 30.16 a*
F_2_(−)	619.90 ± 31.27 a	401.40 ± 20.67 a*

Notes: The values are presented as the mean ± standard errors of the means (SEs). Asterisks indicate significant differences between control and high-temperature treatment materials (* for *p* < 0.05). Different lowercase letters in a column represent statistically significant differences between different materials (*p* < 0.05). GM, genetically modified; NJW, Nanjing wild soybean; F_2_(+), F_2_ plants with foreign genes; F_2_(−), F_2_ plants negative for foreign genes.

**Table 3:** Effects of high temperatures on the number of seeds and full seeds per plant of GM, wild, and hybrid soybeans.

Material	Number of Seeds		Full Seed Number	
	Control	High Temperature	Control	High Temperature
GM	81.30 ± 5.31 b	46.40 ± 6.32 c*	73.30 ± 16.30 b	41.40 ± 11.32 c*
NJW	1096.5 ± 72.54 a	838.60 ± 112.09 a	919.70 ± 231.50 a	629.20 ± 307.67 a
F_2_(+)	944.30 ± 95.85 a	447.50 ± 43.23 b*	684.6 ± 278.65 a	157.2 ± 99.45 b*
F_2_(−)	919.50 ± 36.36 a	520.20 ± 36.38 a*	724.80 ± 87.42 a	264.90 ± 229.30 b*

Notes: The values are presented as the mean ± standard errors of the means (SEs). Asterisks indicate significant differences between control and high-temperature treatment materials (* for *p* < 0.05). Different lowercase letters in a column represent statistically significant differences between different materials (*p* < 0.05). GM, genetically modified; NJW, Nanjing wild soybean; F_2_(+), F_2_ plants with foreign genes; F_2_(−), F_2_ plants negative for foreign genes.

**Table 4:** Effects of high temperature on the hundred-seed weight and seed weight per plant of GM, wild, and hybrid soybeans.

Material	100-Seed Weight (g)		Seed Weight (g)	
	Control	High Temperature	Control	High Temperature
GM	19.11 ± 0.58 a	12.61 ± 0.37 a*	18.48 ± 1.42 c	9.55 ± 0.66 b*
NJW	1.91 ± 0.02 c	1.68 ± 0.04 c*	31.27 ± 2.91 b	17.47 ± 2.01 ab*
F_2_(+)	5.47 ± 0.11 b	3.05 ± 0.17 b*	45.32 ± 2.74 a	22.52 ± 1.56 a*
F_2_(−)	5.21 ± 0.06 b	3.11 ± 0.26 b*	47.66 ± 5.98 a	22.46 ± 2.80 a*

Note: The values are presented as the mean ± standard errors of the means (SEs). Asterisks indicate significant differences between control and high-temperature treatment materials (* for *p* < 0.05). Different lowercase letters in a column represent statistically significant differences between different materials (*p* < 0.05). GM, genetically modified; NJW, Nanjing wild soybean; F_2_(+), F_2_ plants with foreign genes; F_2_(−), F_2_ plants negative for foreign genes.

**Table 5:** Effects of high temperature on the viability of GM, wild, and hybrid soybean seeds.

Material	Seed Germination Rates (%)		Dead Seeds (%)	
	Control	High Temperature	Control	High Temperature
GM	86.5 ± 1.26 b	46.50 ± 7.14 c*	13.50 ± 1.26 a	53.50 ± 7.14 a*
NJW	94.5 ± 0.96 a	84.50 ± 2.75 a*	5.50 ± 0.96 b	15.5 ± 2.75 c*
F_2_(+)	89.00 ± 1.73 ab	58.00 ± 6.32 bc*	11.00 ± 1.73 ab	42.00 ± 6.32 ab*
F_2_(−)	86.50 ± 2.50 b	72.50 ± 4.35 ab*	13.50 ± 2.50 a	27.5 ± 4.35 bc*

Notes: Data are presented as the mean ± standard errors of the means (SEs). Asterisks indicate significant differences between control and high-temperature treatments (* for *p* < 0.05). Different lowercase letters in a column represent statistically significant differences between different materials (*p* < 0.05). GM, genetically modified; NJW, Nanjing wild soybean; F_2_(+), F_2_ plants with foreign genes; F_2_(−), F_2_ plants negative for foreign genes.

## Data Availability

The data collected in this study are available upon request. The data are not publicly available due to institutional restrictions.

## Supplementary Materials

The following supporting information can be downloaded at: https://www.mdpi.com/article/10.3390/plants14040622/s1, Figure S1: Analysis of PAT (A,B) and Vip3Aa (C,D) proteins in soybean plants using colloidal gold immunochromatographic assay test strips (N, negative control; P, positive control; 1-176, F_2_ progenies; C, control line; T, test line); Figure S2: Effects of high temperatures on seed morphology. Range of phenotypes observed in mature seeds: (A) fully filled seeds, (B) partially filled seeds, and (C) seed coat cracks.

## References

[1] ISAAA (International Agricultural Biotechnology Application Service Organization) (2021). Global Biotechnology/GM Crop Commercialization Development Trend in 2019. China Bioeng. J..

[2] Machado E.P., Rodrigues Junior G.L.S., Somavilla J.C., Führ F.M., Zago S.L., Marques L.H., Santos A.C., Nowatzki T., Dahmer M.L., Omoto C. (2020). Survival and Development of *Spodoptera eridania*, *Spodoptera cosmioides* and *Spodoptera albula* (Lepidoptera: Noctuidae) on Genetically Modified Soybean Expressing Cry1Ac and Cry1F Proteins. Pest. Manag. Sci..

[3] Globus R., Qimron U. (2018). A Technological and Regulatory Outlook on CRISPR Crop Editing. J. Cell Biochem..

[4] Pental D. (2019). When Scientists Turn Against Science: Exceptionally Flawed Analysis of Plant Breeding Technologies. Curr. Sci..

[5] Zhang L., Shen W.J., Fang Z.X., Liu B. (2021). Effects of Genetically Modified Maize Expressing Cry1Ab and EPSPS Proteins on Japanese Quail. Poultry Sci..

[6] Fei X., Huang X., Li Z., Li X., He C., Xiao S., Li Y., Zhang X., Deng X. (2023). Effect of Marker-Free Transgenic Chlamydomonas on the Control of *Aedes* Mosquito Population and on Plankton. Parasit. Vectors.

[7] García M.J., Palma-Bautista C., Vazquez-Garcia J.G., Rojano-Delgado A.M., Osuna M.D., Torra J., De P.R. (2020). Multiple Mutations in the EPSPS and ALS Genes of Amaranthus Hybridus Underlie Resistance to Glyphosate and ALS Inhibitors. Sci. Rep..

[8] Zhang L., Liu L., Fang Z., Shen W., Dai Y., Jia R.Z., Liang J.G., Liu B. (2023). Fitness Changes in Wild Soybean Caused by Gene Flow from Genetically Modified Soybean. BMC Plant Biol..

[9] Liu L., Zhang L., Fang Z., Shen W.J., Yin X., Ren Z.T., Yu Q., Liang J.G., Liu B. (2024). Glyphosate Resistance and No Fitness Cost in Backcross Offspring of Wild Soybean and Transgenic Soybean with *Epsps* Gene. BMC Plant Biol..

[10] Tian B., Talukder S.K., Fu J., Fritz A.K., Trick H.N. (2018). Expression of a Rice Soluble Starch Synthase Gene in Transgenic Wheat Improves the Grain Yield under Heat Stress Conditions. Vitr. Cell Dev. Biol. Plant.

[11] Hailemariam H.M. (2022). Adaptability and Stability for Soybean Yield by AMMI and GGE Models in Ethiopia. Front. Plant Sci..

[12] Li W., Liu M., Lai Y.C., Liu J.X., Fan C., Yang G., Wang L., Liang W.W., Di S.F., Yu D.Y. (2022). Genome-Wide Association Study of Partial Resistance to *P. sojae* in Wild Soybeans from Heilongjiang Province, China. Curr. Issues Mol. Biol..

[13] Wang K.J., Li X.H. (2013). Genetic Diversity and Gene Flow Dynamics Revealed in the Rare Mixed Populations of Wild Soybean (*Glycine soja*) and Semi-Wild Type (*Glycine gracilis*) in China. Genet. Resour. Crop Evol..

[14] Zeng Q.Y., Yang C.Y., Ma Q.B., Li X.P., Dong W.W., Nian H. (2012). Identification of Wild Soybean Mirnas and Their Target Genes Responsive to Aluminum Stress. BMC Plant Biol..

[15] Clark M., Maselko M. (2020). Transgene Biocontainment Strategies for Molecular Farming. Front. Plant Sci..

[16] Sohn S.I., Pandian S., Oh Y.J., Kang H.J., Ryu T.H., Cho W.S., Shin E.K., Shin K.S. (2021). A Review of the Unintentional Release of Feral Genetically Modified Rapeseed into the Environment. Biology.

[17] Zhou Z., Zhang C., Lu X., Wang L., Hao Z., Li M., Zhang D., Yong H., Zhu H., Weng J. (2018). Dissecting the Genetic Basis Underlying Combining Ability of Plant Height Related Traits in Maize. Front. Plant Sci..

[18] Xu B., Wu R., Shi F., Gao C., Wang J. (2022). Transcriptome Profiling of Flower Buds of Male-Sterile Lines Provides New Insights into Male Sterility Mechanism in Alfalfa. BMC Plant Biol..

[19] Lu B.R., Xia H. (2011). Environmental Biosafety of Transgenic Plants: Research and Assessment of Transgene Escape and Its Potential Ecological Impacts. Chin. Sci. Bull..

[20] Sohn S.I., Thamilarasan S.K., Pandian S., Oh Y.J., Ryu T.H., Lee G.S., Shin E.K. (2022). Interspecific Hybridization of Transgenic *Brassica napus* and *Brassica rapa*—An Overview. Genes.

[21] Yook M.J., Park H.R., Zhang C.J., Lim S.H., Kim D.S. (2020). Environmental Risk Assessment of Glufosinate-Resistant Soybean by Pollen-Mediated Gene Flow under Field Conditions in the Region of the Genetic Origin. Sci. Total Environ..

[22] Guan Z.J., Zhang P., Wei W., Mi X.C., Kang D.M., Liu B. (2015). Performance of Hybrid Progeny Formed Between Genetically Modified Herbicide-Tolerant Soybean and Its Wild Ancestor. AoB Plants.

[23] Liu L.L., Zhang L., Fu J.M., Shen W.J., Fang Z.X., Dai Y., Jia R.Z., Liu B., Liang J.G. (2022). Fitness and Ecological Risk of Hybrid Progenies of Wild and Herbicide-Tolerant Soybeans with *Epsps* Gene. Front. Plant Sci..

[24] Mimić G., Brdar S., Brkić M., Panić M., Marko O., Crnojević V. (2020). Engineering Meteorological Features to Select Stress Tolerant Hybrids in Maize. Sci. Rep..

[25] Zandalinas S.I., Fritschi F.B., Mittler R. (2021). Global Warming, Climate Change, and Environmental Pollution: Recipe for a Multifactorial Stress Combination Disaster. Trends Plant Sci..

[26] Wang Q., Li X., Qiu Z., Yang S., Zhou W., Zhao J. (2022). Depth-Keeping Control for a Deep-Sea Self-Holding Intelligent Buoy System Based on Inversion Time Constraint Stability Strategy Optimization. Sensors.

[27] Wheeler T.R., Craufurd P.Q., Ellis R.H., Porter J.R., Prasad P.V.V. (2000). Temperature Variability and The Yield of Annual Crops. Agric. Ecosyst. Environ..

[28] Allen L.H., Zhang L., Boote K.J., Hauser B.A. (2018). Elevated Temperature Intensity, Timing, and Duration of Exposure Affect Soybean Internode Elongation, Mainstem Node Number, and Pod Number Per Plant. Crop J..

[29] Djanaguiraman M., Schapaugh W., Fritschi F., Nguyen H., Vara P.P.V. (2019). Reproductive Success of Soybean (*Glycine max* L. Merril) Cultivars and Exotic Lines under High Daytime Temperature. Plant Cell Environ..

[30] Das A., Rushton P.J., Rohila J.S. (2017). Metabolomic Profiling of Soybeans (*Glycine max* L.) Reveals the Importance of Sugar and Nitrogen Metabolism under Drought and Heat Stress. Plants.

[31] Zhang G., Bahn S.C., Wang G., Zhang Y., Chen B., Zhang Y., Wang X., Zhao J. (2019). PLDα1-knockdown Soybean Seeds Display Higher Unsaturated Glycerolipid Contents and Seed Vigor in High Temperature and Humidity Environments. Biotechnol. Biofuels.

[32] Narayanan S., Zoong-Lwe Z.S., Gandhi N., Welti R., Fallen B., Smith J.R., Rustgi S. (2020). Comparative Lipidomic Analysis Reveals Heat Stress Responses of Two Soybean Genotypes Differing in Temperature Sensitivity. Plants.

[33] Lippmann R., Babben S., Menger A., Delker C., Quint M. (2022). Development of Wild and Cultivated Plants under Global Warming Conditions. Curr. Biol..

[34] Zhang J.L., Wang J.X., Wei J., Liu J.G., Yang S.N., Gai J.Y., Li Y. (2016). Identification and Analysis of NaHCO_3_ Stress Responsive Genes in Wild Soybean (*Glycine soja*) Roots by RNA-seq. Front. Plant Sci..

[35] Zhang J., Yang D., Li M., Shi L.X. (2016). Metabolic Profiles Reveal Changes in Wild and Cultivated Soybean Seedling Leaves under Salt Stress. PLoS ONE.

[36] Fu H., Guo R., Shen W.Y., Li M.X., Shi L.X. (2020). Changes in the Metabolome of Two Soybean Genotypes under Drought Stress. Russian J. Plant Physl..

[37] Veremeichik G.N., Brodovskaya E.V., Grigorchuk V.P., Butovets E.S., Lukyanchuk L.M., Bulgakov V.P. (2022). ABA-Dependent Regulation of Calcium-Dependent Protein Kinase Gene *GmCDPK*5 in Cultivated and Wild Soybeans. Life.

[38] Zhao Y., Deng H., Yu C., Hu R. (2019). The Chinese Public’s Awareness and Attitudes Toward Genetically Modified Foods with Different Labeling. NPJ Sci. Food.

[39] Cai J., Hu R., Huang J., Wang X. (2016). Investment, Research Ability and Progress of Agricultural Biotechnology in China. Chin. Rural. Econ..

[40] Jin Y., Schaub S., Tosun J., Wesseler J. (2022). Does China Have a Public Debate on Genetically Modified Organisms? A Discourse Network Analysis of Public Debate on Weibo. Public. Underst. Sci..

[41] Xiang S., Wang S., Xu M., Wang W., Liu W. (2023). YOLO POD: A Fast and Accurate Multi-Task Model for Dense Soybean Pod Counting. Plant Methods.

[42] Guo J., Wang Z., Qu L., Hu Y., Lu D. (2022). Transcriptomic and Alternative Splicing Analyses Provide Insights into the Roles of Exogenous Salicylic Acid Ameliorating Waxy Maize Seedling Growth under Heat Stress. BMC Plant Biol..

[43] Hu X., Wei Q., Wu H., Huang Y., Peng X., Han G., Ma Q., Zhao Y. (2022). Identification and Characterization of Heat-Responsive Lncrnas in Maize Inbred Line CM1. BMC Genom..

[44] Huang Y., Wang Y.Y., Qiang S., Song X.L., Dai W.M. (2019). Fitness of F_1_ hybrids Between Stacked Transgenic Rice T1c-19 With Cry1c*/Bar Genes and Weedy Rice. J. Integr. Agric..

[45] Liu J.Y., Sheng Z.W., Hu Y.Q., Liu Q., Qiang S., Song X.L., Liu B. (2021). Fitness of F_1_ Hybrids Between 10 Maternal Wild Soybean Populations and Transgenic Soybean. Transgenic Res..

[46] Zhang L., Jia R.Z., Liu L., Shen W., Fang Z., Zhou B., Liu B. (2023). Seed Coat Colour and Structure Are Related to the Seed Dormancy and Overwintering Ability of Crop-to-Wild Hybrid Soybean. AoB Plants.

[47] Lu B.R. (2008). Transgene Escape from GM Crops and Potential Biosafety Consequences: An Environmental Perspective. Int. Centre. Genet. Eng. Biotechnol. Collect. Biosaf. Rev..

[48] Liu B., Xue K., Liu L.P., Shen W.J., Guo H. (2020). Research on the Gene Flow from Transgenic EPSPS + PAT Soybean S4003.14 to Non-Transgenic Soybeans. J. Ecol. Rural. Environ..

[49] Hegland S.J., Nielsen A., Lázaro A., Bjerknes A.L., Totland Ø. (2009). How Does Climate Warming Affect Plant-Pollinator Interactions?. Ecol. Lett..

[50] Wang C.H., Li B. (2016). Salinity and Disturbance Mediate Direct and Indirect Plant-Plant Interactions in An Assembled Marsh Community. Oecologia.

[51] Du L., Qu M., Jiang X., Li X., Ju Q., Lu X., Wang J. (2019). Fitness Costs Associated with Acetyl-Coenzyme A Carboxylase Mutations Endowing Herbicide Resistance in *American sloughgrass* (*Beckmannia syzigachne* Steud.). Ecol. Evol..

[52] Snow A.A., Pilson D., Rieseberg L.H., Paulsen M.J., Pleskac N., Reagon M.R., Wolf D.E., Selbo S.M. (2003). A Bt Transgene Reduces Herbivory and Enhances Fecundity in Wild Sunflowers. Ecol. Appl..

[53] Rinella M.J., Pokorny M.L., Rekaya R. (2007). Grassland Invader Responses to Realistic Changes in Native Species Richness. Ecol. Appl..

[54] Di K., Stewart C.N., Wei W., Shen B.C., Tang Z.X., Ma K.P. (2009). Fitness and Maternal Effects in Hybrids Formed Between Transgenic Oilseed Rape (*Brassica napus* L.) and Wild Brown Mustard [*B. juncea* (L.) Czern et Coss.] in the Field. Pest. Manag. Sci..

[55] Song X., Wang Z., Qiang S. (2011). Agronomic Performance of F_1_, F_2_ And F_3_ Hybrids Between Weedy Rice and Transgenic Glufosinate-Resistant Rice. Pest. Manag. Sci..

[56] Hinojosa L., Matanguihan J.B., Murphy K.M. (2019). Effect of High Temperature on Pollen Morphology, Plant Growth and Seed Yield in Quinoa (*Chenopodium quinoa* Willd). J. Agron. Crop Sci..

[57] Raja M.M., Vijayalakshmi G., Naik M.L., Basha P.O., Sergeant K., Hausman J.F., Khan P.S.S.V. (2019). Pollen Development and Function under Heat Stress: From Effects to Responses. Acta Physiol. Plant..

[58] Liang R., Ji X., Sheng Z., Liu J., Qiang S., Song X. (2022). Fitness and Rhizobacteria of F_2_, F_3_ Hybrids of Herbicide-Tolerant Transgenic Soybean and Wild Soybean. Plants.

[59] Sehgal A., Sita K., Kumar J., Kumar S., Singh S., Siddique K.H.M., Nayyar H. (2017). Effects of Drought, Heat and Their Interaction on the Growth, Yield and Photosynthetic Function of Lentil (*Lens culinaris* Medikus) Genotypes Varying in Heat and Drought Sensitivity. Front. Plant Sci..

[60] Adeyemi N.O., Atayese M.O., Olubode A.A., Akan M.E. (2020). Effect of Commercial Arbuscular Mycorrhizal Fungi Inoculant on Growth and Yield of Soybean under Controlled and Natural Field Conditions. J. Plant Nutr..

[61] Tang Y., Lu S., Fang C., Liu H., Dong L., Li H., Su T., Li S., Wang L., Cheng Q. (2023). Diverse Flowering Responses Subjecting to Ambient High Temperature in Soybean under Short-Day Conditions. Plant Biotechnol. J..

[62] Trtikova M., Wikmark O.G., Zemp N., Widmer A., Hilbeck A. (2015). Transgene Expression and Bt Protein Content in Transgenic Bt maize (MON810) under Optimal and Stressful Environmental Conditions. PLoS ONE.

[63] Chen Y., Chen Y.J., Chen Y., Zhang X., Wang Y.H., Chen D.H. (2013). The Recovery of Bt Toxin Content After Temperature Stress Termination in Transgenic Cotton. Span. J. Agric. Res..

[64] Zhang X., Rui Q.Z., Liang P.P., Wei C.H., Deng G.Q., Chen Y., Dong Z.D., Chen D.H. (2018). Dynamics of Bt Cotton Cry1Ac protein Content under an Alternating High Temperature Regime and Effects on Nitrogen Metabolism. J. Integr. Agric..

[65] Feng L., Zhu S., Liu F., He Y., Bao Y., Zhang C. (2019). Hyperspectral Imaging for Seed Quality and Safety Inspection: A Review. Plant Methods.

[66] Shu Y., Zhou Y., Mu K., Hu H., Chen M., He Q., Huang S., Ma H., Yu X. (2020). A Transcriptomic Analysis Reveals Soybean Seed Pre-Harvest Deterioration Resistance Pathways under High Temperature and Humidity Stress. Genome.

[67] Liu S., Liu Y., Liu C., Li Y., Zhang F., Ma H. (2022). Isolation and Characterization of the GmMT-II Gene and Its Role in Response to High Temperature and Humidity Stress in *Glycine max*. Plants.

[68] Musielak G. (2000). Influence of the Drying Medium Parameters on Drying Induced Stresses. Dry. Technol..

[69] Igathinathane C., Chattopadhyay P.K., Pordesimo L.O. (2008). Moisture Diffusion Modeling of Parboiled Paddy Accelerated Tempering Process with Extended Application to Multi-Pass Drying Simulation. J. Food Eng..

[70] Liu F., Li N., Yu Y., Chen W., Yu S., He H. (2022). Insights into the Regulation of Rice Seed Storability by Seed Tissue-Specific Transcriptomic and Metabolic Profiling. Plants.

[71] Ma F., Ewa C., Tasneem M., Carol A.P., Mark G. (2004). Cracks in The Palisade Cuticle of Soybean Seed Coats Correlate with Their Permeability to Water. Ann. Bot-London.

[72] Santos E.L.D., José Nivaldo P., Barros A.S.D.R., Prete C.E.C. (2007). Soybean Seed Coat Variation and Its Influence on the Physiological Quality and Chemical Composition. Rev. Bras. De Sementes.

[73] Nurzyńska-Wierdak R., Łabuda H., Buczkowska H., Sałata A. (2019). Pericarp of Colored-Seeded Common Bean (*Phaseolus vulgaris* L.) Varieties a Potential Source of Polyphenolic Compounds. Agron. Res..

[74] Zhou S., Sekizaki H., Yang Z., Sawa S., Pan J. (2010). Phenolics in the Seed Coat of Wild Soybean (*Glycine soja*) and Their Significance for Seed Hardness and Seed Germination. J. Agric. Food Chem..

[75] Malenčić D., Cvejić J., Miladinović J. (2012). Polyphenol Content and Antioxidant Properties of Colored Soybean Seeds from Central Europe. J. Med. Food.

[76] Wang M., Gillaspie A., Morris J., Pittman R., Davis J., Pederson G. (2008). Flavonoid Content in Different Legume Germplasm Seeds Quantified by HPLC. Plant Genet. Resour.-C.

[77] Ren Z., Yin X., Liu L., Zhang L., Shen W., Fang Z., Yu Q., Qin L., Chen L., Jia R. (2024). Flavonoid Localization in Soybean Seeds: Comparative Analysis of Wild (*Glycine soja*) and Cultivated (*Glycine max*) Varieties. Food Chem..

[78] Hinman E.D., Fridley J.D., Parry D. (2019). Plant Defense Against Generalist Herbivores in the Forest Understory: A Phylogenetic Comparison of Native and Invasive Species. Biol. Invasions.

[79] Gompert Z., Fordyce J.A., Forister M.L., Shapiro A.M., Nice C.C. (2006). Homoploid Hybrid Speciation in An Extreme Habitat. Science.

[80] Xiang D., Luo M., Jiang F., Wen Z., Chen X., Wang X., Xu X., Wei W., Xu J. (2023). Safety Assessment of Subchronic Feeding of Insect-Resistant and Herbicide-Resistant Transgenic Soybeans to Juvenile Channel Catfish (*Ictalurus punctatus*). Sci. Rep..

[81] Fehr W.R., Caviness C.E., Burmood D.T., Pennington J.S. (1971). Stage of Development Description for Soybeans, *Glycine max* (L.) Merrill1. Crop Sci..

[82] Djanaguiraman M., Prasad P.V.V., Boyle D.L., Schapaugh W.T. (2013). Soybean Pollen Anatomy, Viability and Pod Set under High Temperature Stress. J. Agron. Crop Sci..

