# Supplementary figures and images for: Changes in the Stress Response and Fitness of Hybrids Between Transgenic Soybean and Wild-Type Plants Under Heat Stress
Source: Plants (Basel). 2025 Feb 19;14(4):622. doi: 10.3390/plants14040622 (PMC11860058; doi:10.3390/plants14040622)

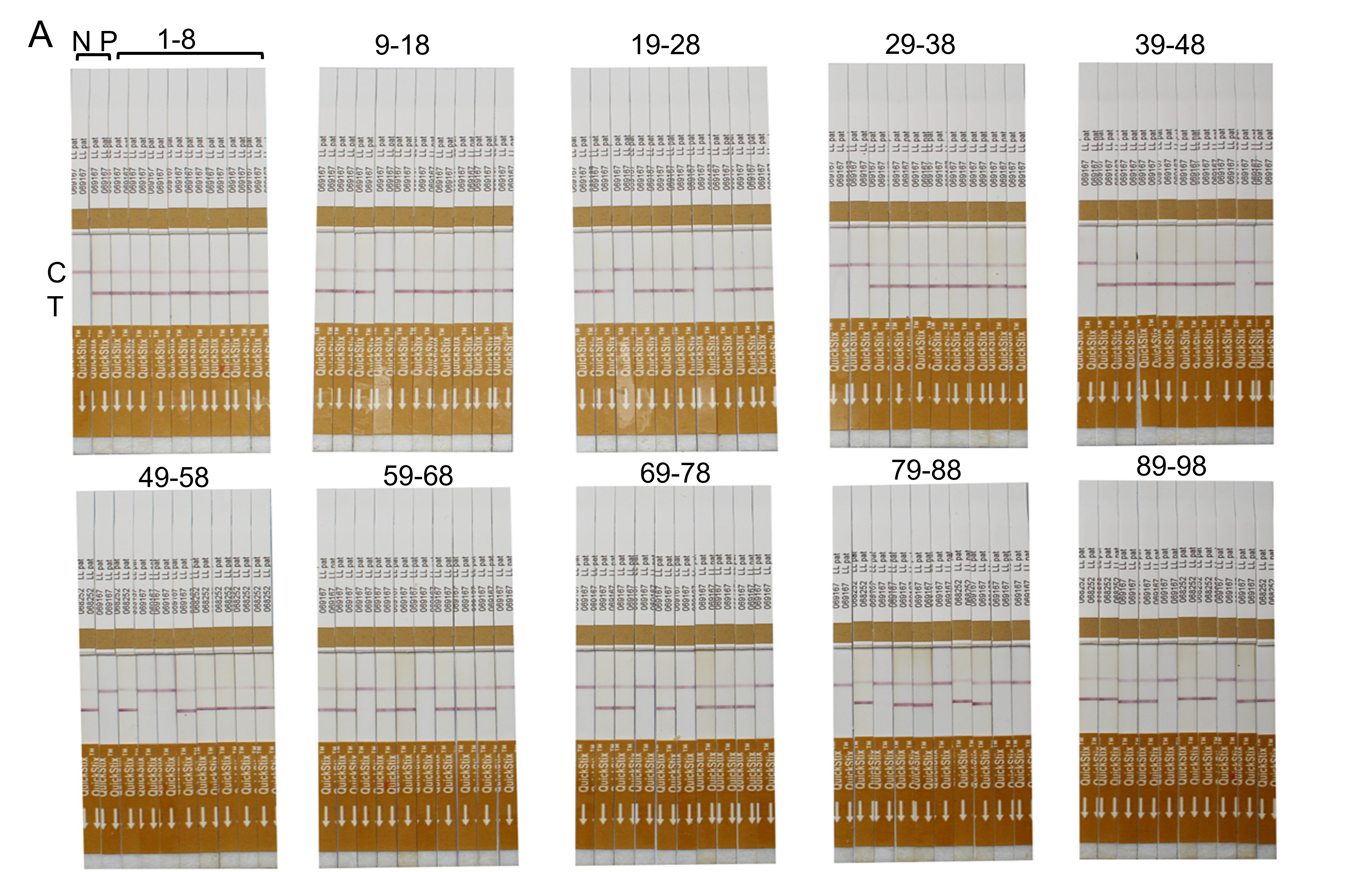

Supplement: Supplementary file 1 [file plants-14-00622-s001.zip › Figure S1-A.tif]

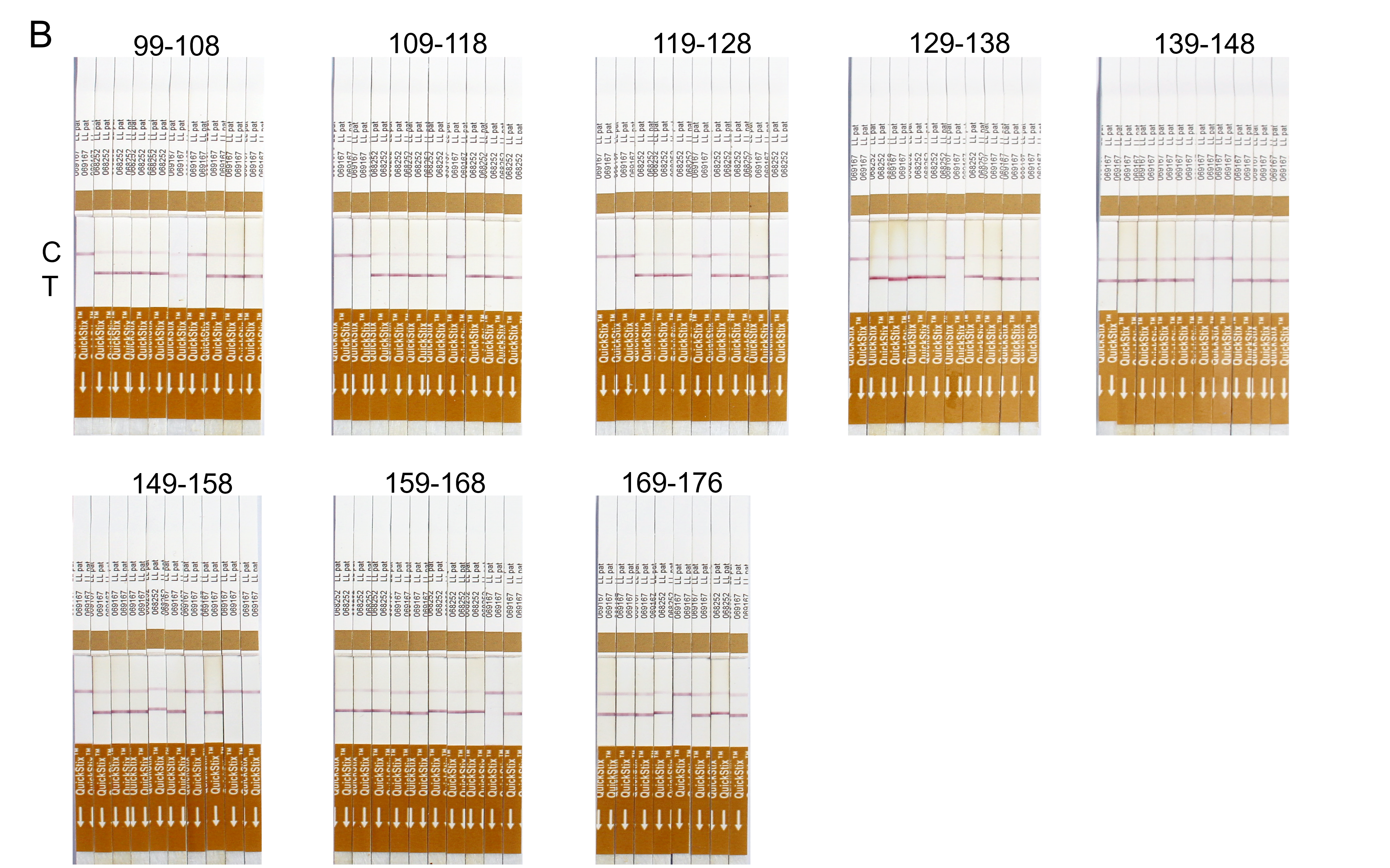

Supplement: Supplementary file 1 [file plants-14-00622-s001.zip › Figure S1-B.tif]

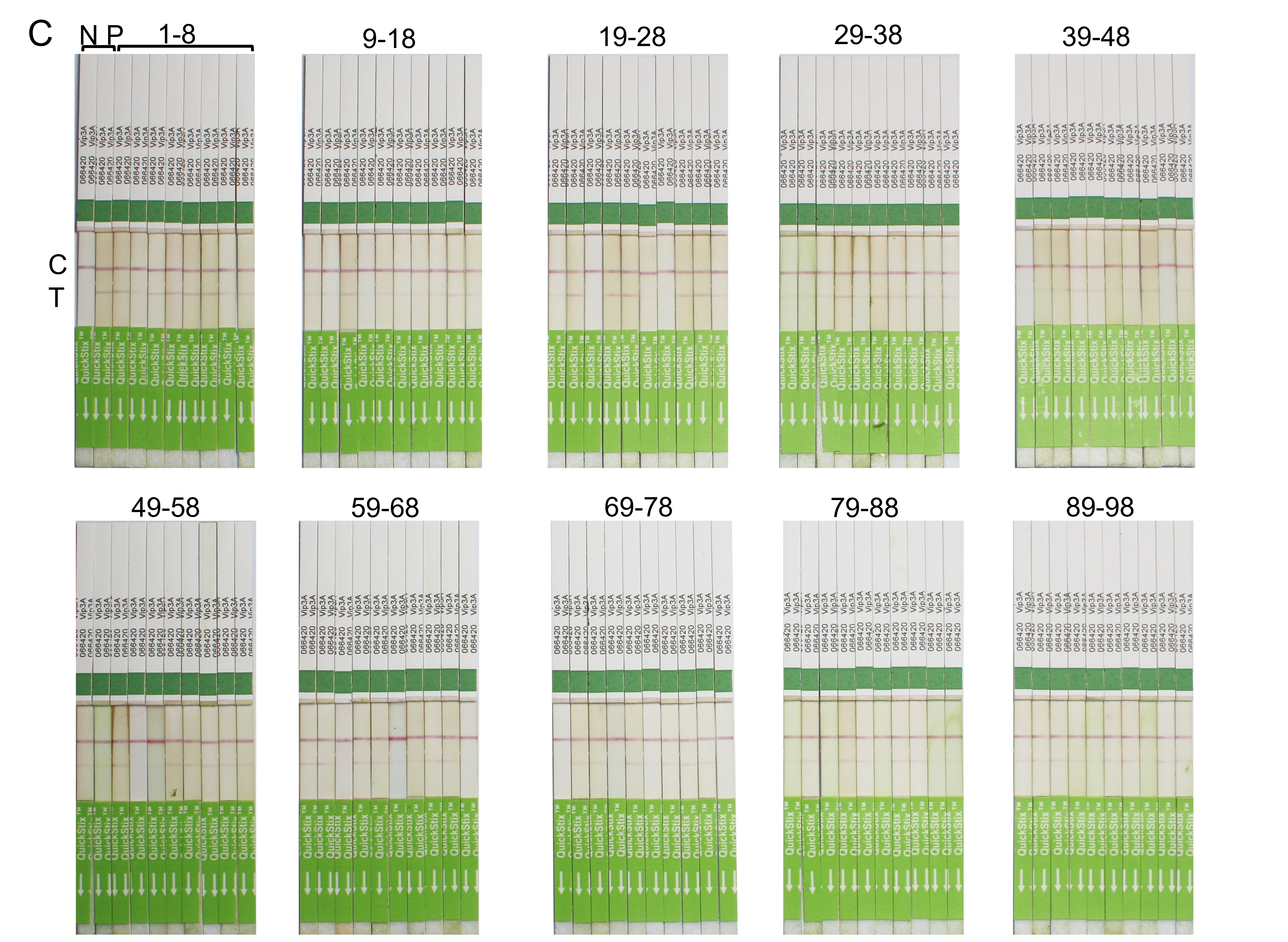

Supplement: Supplementary file 1 [file plants-14-00622-s001.zip › Figure S1-C.tif]

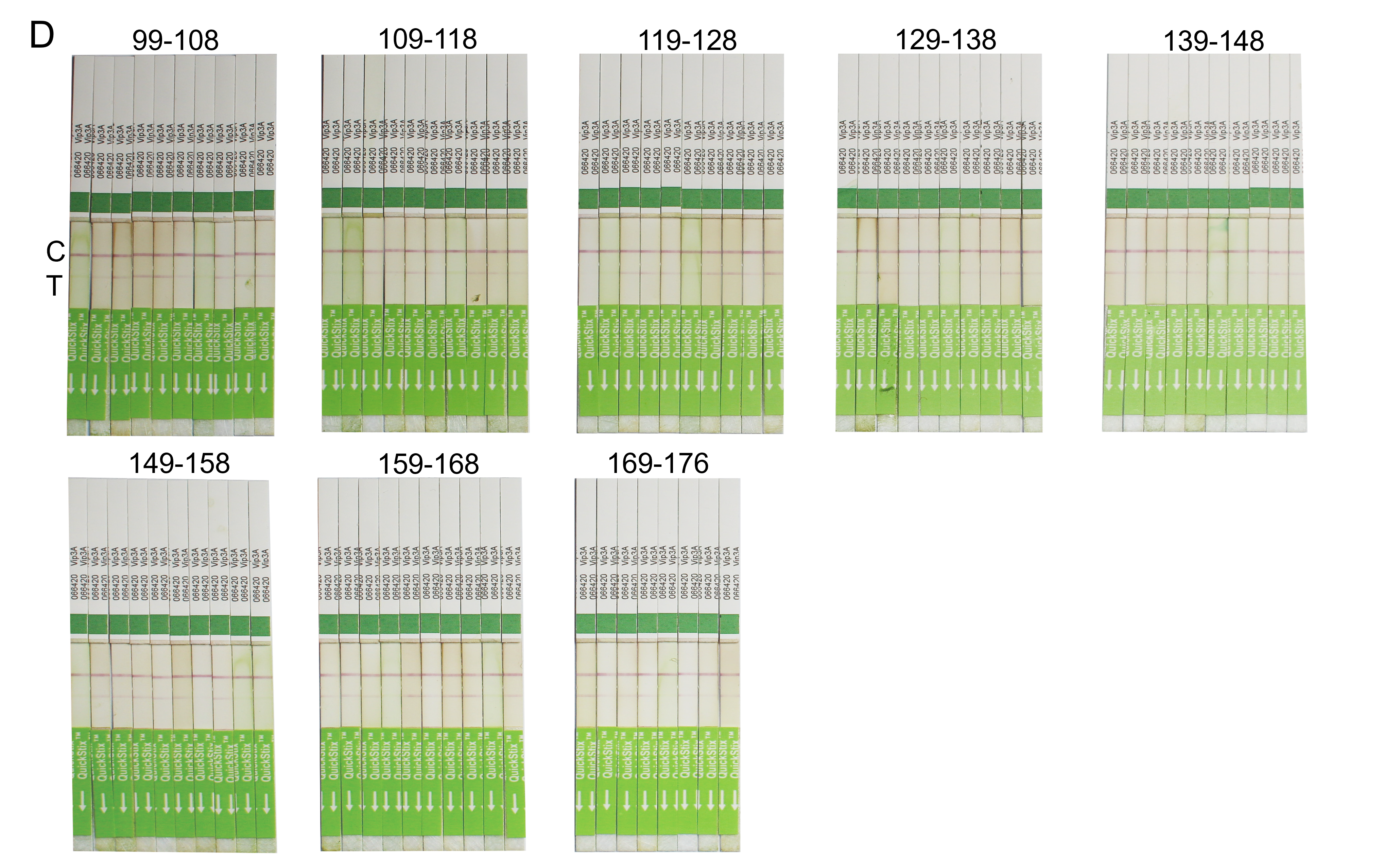

Supplement: Supplementary file 1 [file plants-14-00622-s001.zip › Figure S1-D.tif]

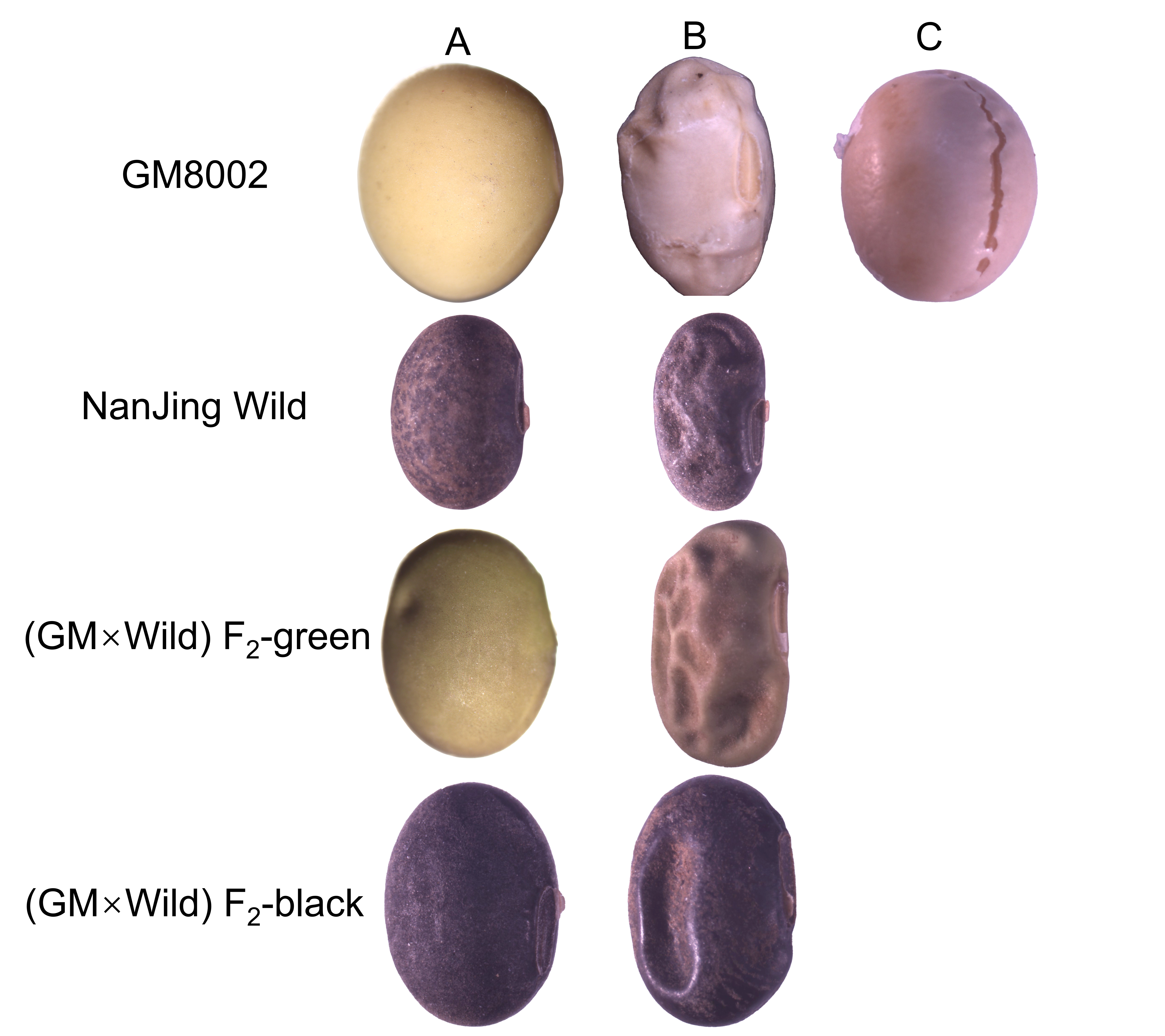

Supplement: Supplementary file 1 [file plants-14-00622-s001.zip › Figure S2.tif]
